# Supplementary material for: Are carbon emissions trading and green financial instruments synergistic? -Comprehensive quantitative research based on content analysis
Source: PLoS One. 2024 Mar 7;19(3):e0298601. doi: 10.1371/journal.pone.0298601 (PMC10919601; doi:10.1371/journal.pone.0298601)
Supplement: S1 Data — (ZIP) [file pone.0298601.s001.zip › Data/地级市Z_F工作报告.zip!/║┌┴·╜¡/┤≤╟∞╩╨/└┤╫╘╣1⁄2╓┌║┼í╛┬φ┐╦╩2╛▌═°í┐_2015.docx]

各位代表：

　　现在，我代表市人民政府向大会报告工作，请予审议，并请各位政协委员和列席会议的同志提出意见。

　　 一、2014年工作回顾

　　过去一年，我们深入贯彻党的十八大和十八届三中、四中全会精神，按照市委八届四次全会要求和市九届人大三次会议部署，深化落实国家和省市各项政策，组合实施“10个百”稳增长举措，努力克服经济下行压力，经济社会持续健康发展。全市完成地区生产总值4070亿元、增长4.5%；地方财政收入254.8亿元、下降2.4%；城镇登记失业率控制在4.2%；城镇居民人均可支配收入32307元、农村居民人均可支配收入12443元，分别增长9%和13.1%；万元gdp综合能耗下降3.3%。

　　（一）经济稳定增长。工业平稳发展，增加值增长3.8%。原油稳产4000万吨，生产天然气35.1亿立方米；15万吨丙烯、170万吨柴油加氢等石化大项目建成投用，乙烯产量突破百万吨，石化产业增加值增长7.1%；沃尔沃汽车批量生产并上市销售，高分子复合材料等66个重点工业项目投产，新增规上工业企业60家，华科、联勤等骨干企业规模扩张，地方工业增加值增长10%；省级以上科技企业孵化器达到5家，在孵企业459家，新获批国家级高新技术企业51家。农业较快发展，增加值增长8.5%。粮食、畜牧、果蔬生产“三位一体”格局持续巩固，粮食产量106.7亿斤，大庆连续三年被评为全国粮食生产先进市；“两牛一猪”存栏264万头、禽类1680万只，规模化养殖比重提高到70%；棚室果蔬产量40亿斤，销售收入56亿元。进入全国黑龙江绿色食品旗舰店的大庆产品136种，开办独立品牌连锁店7家。大庆市被农业部确定为国家现代农业示范区。服务业升级发展，增加值增长8%。红星美凯龙、昌升商贸城、阿木塔景区、中国网库大庆区域平台等项目投用，大庆农商行挂牌运营，浦发银行大庆分行成立，中信银行设立大庆营业部，全市金融机构贷款余额898亿元，新增贷款128亿元，其中实体经济贷款109亿元，鲁冀管业在“新三板”上市、全市上市企业达到22家。旅游、文化创意、服务外包、信息消费、电子商务、健康养老等产业均实现两位数增长，全市社会消费品零售总额增长11.2%。

　　（二）民生持续改善。社会保障更加完善。“五险”净增5.8万人，企业退休人员养老金月人均增加210元，城乡居民基本医保财政补助标准提高到347.5元、比全省标准高8.6%，城镇参保居民政策内医药报销比例达到70%，新农合参合农民政策内医疗费用报销比例达到75%以上；城乡低保标准分别提高19%和44%，农村五保对象集中和分散供养标准分别提高65.6%和60.3%，为19.1万名救助对象发放御寒补助4961万元，扩大低收入家庭认定范围并落实基本生活费等临时救助政策，惠及1.1万人；新增养老床位1038张，“医养结合”模式延伸拓展；改造利民苑等城市老居住区16个，增加保障性住房6010套，改造农村泥草房11500户，2013年洪涝灾害损毁房屋重建全部完成。交通出行更加便利。萨尔图机场新开通航线2条，增加航点城市4个；优化市区公交线路11条，增开线路7条、增设站点26个；按照“十统一”标准推进出租车公司规模化标准化运营，新增出租车1890台、总数达到4415台。教育资源配置更加优化。大庆四中等8所学校新校区建成投用，新增公办公助幼儿园16所，规范民办幼儿园37所，培训幼师3000人次；城乡义务教育标准化学校综合达标率76.2%、提高7个百分点，培训教师2.6万人次；远程教育系统延伸到乡镇学校，全市基础教育“班班通”达到80%，中小学宽带接入率达到77%，优质教育资源覆盖面持续扩大。医疗服务更加健全。市疾控中心改造升级，市妇女儿童医院、龙南医院门诊楼等建成投用，新建乡镇卫生院和村卫生室97个，为乡镇卫生院公开招聘91名医学本专科毕业生，村卫生室运行费用纳入地方财政预算；社区“健康小屋”达到36个，累计体检10万人次；6家市县区医院与解放军301医院等4家名院开展远程会诊，全国百余位知名专家来庆诊治病人近4000人次；面向老年人、孕产妇、残疾人等特需人群的全科医生签约服务逐步推开，签约2万人。文化生活更加丰富。市图书馆、世博会石油馆、大同区文化中心等11个新建成的市县级文体场馆开放运营，建设中心村文化广场71个，482个农家书屋补充图书期刊11万册，城市社区和行政村健身设施覆盖率分别提高到90%和75%以上。国家交响乐团领办的“聆听经典”交响音乐季举办8场演出，高雅艺术进校园演出40场，全市推出文艺精品270余部，全社会唱响大庆幸福谣。社会治理更加扎实。“百湖民声”网络问政平台功能提升，“大庆发布”微博微信政务平台开通；新建改建社区服务综合体20个，乘风社区等社会组织孵化基地投用；覆盖10大类、20个品种的餐饮环节食品安全追溯系统建立运行；各级调解组织调处解决矛盾纠纷19708件，安全生产事故和八类主要刑事案件持续下降，社会保持和谐安定。（三）城乡建设管理扎实推进。基础设施继续完善。实施城建重点工程261项、完工122项。铁路东站普客春节前开通，城际客专东西两站车场建成；改造安萨路、让林路等城乡道路146公里；新增市区管道天然气用户3.2万户，新建改建供热和给排水管网491公里，应急备用水源地建成投用，北城热源与油田热电厂、龙唐供热联通，实现应急情况下统一调控；新建和改造农村饮水安全工程355处、惠及15.6万人，四县城关镇集中供热普及率、生活垃圾处理率、污水处理率分别达到60%、90%和77%。规划管理继续加强。新编制完成《大庆市域村镇体系规划》，完善物流体系布局等控详规划和专项规划23项；出台《关于进一步加强城市规划建设管理监管工作的若干意见》，加强审计监督，规范监管行为，整治工程建设领域突出问题；治理城乡环境，拆除违法违规建筑2000余处、5.5万平方米，补充加密市区重要节点监控探头3000个，新增大型环卫和清冰雪设备49台套、机械化作业率提高到75%。生态环境继续优化。新增城区绿化面积837公顷，绿化升级城区道路119条，建成区绿化覆盖率45.4%；农村造林12.1万亩，全市有林面积达到400万亩；综合治理滨洲湖等湖泊6个，淘汰黄标车1.7万辆、燃煤小锅炉151台，开展野外焚烧秸秆等专项整治，空气质量保持较高水平。大庆在全省率先成为省级生态市。

　　（四）改革开放呈现新活力。重点领域改革深入推进。调整下放市级行政审批事项277项，减少合并审批环节95个，同步调整下放行政审批以外市级行政权力1206项，并针对事权承接组织市里相关部门与县区对口培训800多人次；改革食品药品监管和工商质监管理体制，整合组建市食品药品监督管理局和县区市场监督管理局；落实“先照后证”等措施促进工商注册制度便利化，新注册各类市场主体2.9万户，其中公司制企业3800户、增长68%；推动土地经营权流转，规模以上新型经营主体发展到2630个，拓宽涉农抵押担保范围，建立试点信贷平台，完成农信社改制；启动4所县级公立医院国家改革试点，取消药品加成，实行基本药物零差价销售；社区管理体制改革由市区向县域拓展，四县32个城镇社区整合为18个，基层管理得到加强。对外交流合作取得成效。俄罗斯、马来西亚、港澳台等国家和地区的30多个团组来庆考察和项目对接，全年引进亿元以上项目74个，到位资金319亿元；中科创业园入驻企业226家，总部集聚效应显现；龙泰绿色食品等品牌打入香港市场，机电、石蜡等产品对俄出口规模扩大，全市外贸进出口总额增长20%。政府部门作风进一步改进。扎实开展党的群众路线教育实践活动，认真开展批评与自我批评，着力解决群众关切的现实问题，服务基层、服务群众意识增强；突出问题导向，查摆整改“四风”问题，查补制度漏洞，健全长效机制，保障作风持续改进；严格执行中央八项规定，认真遵守公务接待、差旅费和出国境管理等相关规定，市本级“三公”经费支出减少30%、市政府召开的全市性工作会议减少22%、文件简报减少40%；强化行政效能监察，成立市企业投诉中心，受理各类投诉事项323件、办结率93.5%。

　　国防动员、广播电视、新闻出版、民族宗教、外事侨务、人口计生、防灾减灾、应急管理、统计监测等项工作，妇女儿童、残疾人、慈善和老龄等项事业，都取得了新成绩。

　　各位代表，过去一年全市经济社会发展取得的成果来之不易，这得益于市委的统一领导，得益于人大、政协的有力监督，得益于各民主党派、工商联、无党派人士和群团组织的积极参与，得益于石油石化大企业和中省直单位的鼎力支持，得益于老领导老同志的关心帮助，得益于全市广大干部群众的团结奋斗。在此，我代表市政府，向全市人民，向所有为大庆发展做出贡献的同志们、朋友们，向驻庆部队和武警官兵，表示崇高的敬意和衷心的感谢！

　　在总结工作成绩的同时，我们也清醒地认识到存在的差距和问题。主要是：由于原油价格大幅下降，经济增速明显放缓，结构性矛盾进一步凸显，转型发展的任务更为艰巨；城乡二元结构明显，农民收入相对不高，农村公共服务体系不够完善，民生改善的整体性和均衡性有待增强；事关群众切身利益的一些热点难点问题还比较突出，维护社会和谐稳定的任务繁重；有些政府部门和公务人员服务意识、规则意识、法治意识不强，依法行政能力水平与发展要求不相适应。对这些问题，我们要在今后工作中认真解决。 二、2015年工作安排

　　今年大庆经济发展出现历史拐点，我们对形势必须有清醒判断和理性认识。

　　从发展压力看：稳增长的压力大，受全国“三期叠加”、经济下行等因素影响，大庆同其他资源型城市一样面临着经济稳增长的现实挑战，特别是大庆原油产量从今年开始调减150万吨，而且整个“十三五”期间平均每年都要减产130多万吨，加之目前油价处于低位，给全市经济总量带来较大影响；结构调整的压力大，地方规上工业占规上工业比重18.3%，服务业占全市经济比重18.5%，非公经济占比25%，经济外向度6%左右，调结构转方式任重道远；财政收入的压力大，原油“量价双降”给地方财政收入带来冲击，房地产市场持续低迷影响基金收入，加之结构性减税等因素综合作用导致财政收入趋紧；民生保障的压力大，经济增速放缓、财政增收遇到困难，势必在经济社会各个领域产生传导效应，也给扩大就业、增加收入、社会救助、维护稳定等民生事业发展带来新的挑战。

　　从发展机遇看：国家支持东北振兴和资源型城市转型发展的政策力度加大，我省深入实施“五大规划”、加快建设“中蒙俄经济走廊”龙江陆海丝绸之路经济带、全面推进与中石油签订的战略合作框架协议，中石油在勘探、钻井、化工等领域进一步加大混合所有制经济发展力度，这些都为大庆转型发展提供了更有利的政策环境；原油产量虽然梯度递减但仍处于较高水平，油减气增而且油田公司实施多元发展战略，为今后一个时期稳定全市发展基本面奠定了基础；近三年投产的700多个产业项目陆续投产达效，石油化工、装备制造、汽车制造、食品加工、生物医药、新材料新能源、现代农业和现代服务业等接续产业不断壮大，为产业结构特别是工业结构调整提供了必要支撑；伴随新型城镇化建设，城镇功能提升、人口持续集聚、需求取向多元，为扩大城乡消费、催生产业积蓄了更多潜能；比较完善的城市基础设施、现代立体交通、园区承载功能，不断提升的教育、医疗、文化等公共服务体系，较为富集的天然气、石化、农牧等优质原料，相对充足的科技、人才、土地等生产要素，为培育经济增长点提供了综合比较优势；经过长期保护涵养，域内江河、湖泊、草原、湿地、温泉等资源禀赋和优良空气环境形成了突出的生态优势，为引入外部需求发展旅游、休闲、健康、养老等服务业创造了条件和空间。我们要组合放大这些有利因素，切实把改革创新和政策机遇的诸多红利释放出来，把不断积累的多元经济潜力释放出来，把业已形成的综合实力释放出来，做到减速不减势，强基利长远。

　　今年政府工作总的要求是，深入贯彻党的十八大和十八届三中、四中全会精神，按照市委八届六次全会部署，主动适应经济发展新常态，以转型发展为引领，坚持稳中求进总基调，突出改善民生首要目标，强化改革创新驱动，推进法治政府建设，确保社会和谐稳定，全力推动大庆可持续发展。主要预期目标：地区生产总值增长1.5%左右，其中非油经济增加值增长6%，城镇登记失业率控制在4.5%以内，城镇和农村居民人均可支配收入分别增长10%和12%。

　　今年发展主要预期目标权衡了各方面因素，契合经济发展新常态和我市经济发展阶段性特征。1.5%左右增速体现稳中有进，是在原油产量和价格“双降”负向拉动情况下实现的增长，是要靠非油经济增量把下降部分“填平补齐”再拉动实现的增长，是在4000多亿元较大基数上实现的增长；1.5%左右增速具有合理性，能够守住经济增长底线并给结构调整留有余地，能够稳定就业、稳定居民收入，能够为教育、医疗、文化、社保等公共事业提供保障；1.5%左右增速是全市包括油在内的预期性指标，各县区和高新区不能简单比照，而是要对照全市地方经济增长预期确定科学合理的发展目标。

　　实现全年发展的主要预期目标，要推动三次产业协调发展，努力“稳中间提两头”，力促非油工业有更大作为稳住二产，做精做绿农业提升一产，做大做活服务业提升三产；要狠抓产业项目建设，通过技术升级、合资合作和数量扩张优化存量，通过招商引入要素、科技成果落地、转化资源优势快上增量，推动效益提升和结构调整；要用足用好稳增长政策，继续推进“10个百”组合措施，统筹促进经济增长和民生改善。

（一）推动工业稳定发展。深化地企合作，夯实油化发展基础。合力落实中石油集团与省政府签订的《深化战略合作框架协议》，倾力服务油田公司原油生产、天然气上产和“四大工程”建设；支持石化公司推进千万吨炼油扩能改造、120万吨甲醇制烯烃项目建设，助力炼化公司谋划千万吨炼油和百万吨聚丙烯改扩建，推动7万吨磺酸盐、15万吨丙烯、2亿立方米天然气制氢等项目投产；促进锡达、三聚能源等地方石化企业与中直石化大企业深化合作，搞好延伸吃配。依托龙头项目，壮大地方重点产业。推动沃尔沃现有车型上产，新建spa可扩展整车生产平台，启动汽车配套产业园，建设江森座椅、李尔线束等配套项目，带动汽车产业形成规模；推动忠旺完成硬合金主体厂房和生产线安装条件建设，佳昌科技、同禹傲思等企业扩大规模，带动新材料产业成长壮大；推动伊利奶粉生产由中间产品向终端产品改造升级，百森、天泰等企业增强实力，带动食品产业做精做大；推动大丰加快三次采油技术服务平台建设，北研、力神等企业开拓国际市场，带动装备制造产业提升层次；推动福瑞邦生物制药项目全面投产，志飞、博润等企业加速壮大，带动生物产业集群发展；推动恒基电池片及组件、光伏电站、远景风力发电机组设备等项目投产，绿源风电、弘扬能源等企业技术升级，带动新能源产业加速扩张；推动中宇制革等项目建设，福兴、诚信等企业开发新产品，带动皮革产业关联发展。全市新增规上工业企业40家以上，地方规上工业增加值增长8%。做强园区经济，打造重要增长板块。高新区突出规模效益，重点集聚高新技术产业、战略新兴产业和现代服务业；经开区合理规划布局，重点发展石油石化装备、新材料和新能源等特色产业；林源工业区和各县区专业园区突出比较优势，重点做大精细化工、食品加工、节能环保等立园产业，全市园区工业增加值增长9.5%。强化科技引领，助推产业升级发展。把科技成果转化、新技术应用、科技型企业培育作为调整工业结构的源头路径，支持装备制造、食品加工等行业技术升级，向价值链高端延伸；支持高校发展与地方产业联系紧密的重点专业和特色学科，拓宽共建渠道促进科研成果市场化；用足用活全省千户科技型企业三年行动计划政策，通过梳理成果、成立公司、进行孵化、与资本市场合作，放大生物医药产业联盟、高新区新兴企业孵化中心等平台作用，力争新培育产值超500万元科技型企业100家，国家级高新技术企业突破130家。

　　（二）促进农业提质增效。坚持优质粮食、安全牧业、绿色果蔬一体发展。转变农业发展方式，突出数量质量效益并重，加快建设国家现代农业示范区，深入实施农业科技“六项工程”，新增高标准农田30万亩、水田20万亩，力争粮食产量110亿斤；建好大同航天果蔬等棚室基地，完善集散批发和物流配送功能，果蔬产量突破45亿斤；持续推进畜牧养殖“三退三进”，新增“两牛一猪”及禽类规模化养殖场90个，规模化养殖比重提高到75%。促进农牧生产、精深加工、市场营销链式发展。围绕延伸黑土玉米、沿江水稻、沙地杂粮、寒地果蔬等原料加工产业链，增强龙头企业带动能力，绿色食品基地达到600万亩，市级以上规模加工企业发展到150家；关联发展涉农服务业，嫁接催生冷链物流、网上交易、观光采摘、礼品农业等新兴业态，加快农业接二产连三产深度融合。推进设施建设、资源开发、生态保护协调发展。抓好水稻育秧基地、新型农机服务体和农业物联网应用平台建设，新建标准化育秧大棚3100栋、智能化催芽车间4个，新增大型农机合作社10个，新增“节水增粮”面积50万亩；依托河湖资源，建好沿江300万亩水稻种植带，大力发展绿色水产养殖业；加强农业面源污染治理，严控化肥和农药施用量，治理“三化”草原10万亩，建设丰产林和经济林5000亩。推动合作化经营、企业化管理、社会化融资共进发展。健全土地流转服务平台，采取股份合作、联营联合、专业合作等方式，在农民自愿的前提下推动土地适度规模经营，新增50万亩以上；鼓励职业农民与非农资本、职业经纪人和新技术充分对接，提升农业生产经营规范化、标准化、市场化水平；通过财政支持、企业投入、农民自筹和工商资本注入等渠道，健全农业融资体系。

　　（三）推进服务业提档升级。商贸物流业支持大商等大型商贸企业实体店营销与电商营销互动发展，推动让胡路鱼街、萨尔图茶城等特色街区建设，扩大便民商业网点覆盖，新增社区连锁店和乡村网购配送点100个以上；抓好沈铁内陆港50万吨仓容、大连百丰农品商贸综合体等项目建设，加快发展仓储、货代、联运等业态，力争引进国内百强物流企业2家以上。旅游业抓好专项规划编制，突出打造雪地温泉游、文博文化游、民族风情游等特色品牌，增强地接服务能力，搞好湿地旅游文化节等主题推介，携手旅游电商做好产品线上营销，联合知名旅行社拓展客源市场。文化产业发挥百湖影视基地、艺术群落和歌剧院等场馆功能，创意催生影视动漫、精品演出、主题会展、文化时尚、大众健身等文化产品，引进中国素质机器人运动项目落户大庆，推动大学生戏剧节、儿童小剧场、汽车影院等市场细分项目的开发，丰富文化产品供给。金融业支持现有银行创新产品，扩大证券、保险、基金业务规模，引进创投风投机构，规范小额贷款公司运营，新增注册资金超亿元融资性民营担保公司2家以上、“新三板”上市企业3家以上，构建金融多元发展格局。信息服务业投用联想云计算中心，启动新华08产交所金融资产及股权交易平台，新增服务外包企业20家以上，新建4g网络基站725个，拓展增值业务，扩大信息消费。健康养老服务业坚持公办引领、民办补充、居家为主，鼓励社会力量兴办养老机构，新增机构养老床位1000张；支持现有厂办医院、二级以上医院植入养老功能，转型发展“医养结合”产业；借助良好的生态化优势，发展候鸟式养老。

（四）整体均衡改善民生。越是经济下行、财力紧张，越要把群众冷暖放在心上，越要把民生福祉抓在手上。民生支出同比增长3.2%，占公共财政支出比重提高到60.1%。创业带动就业，掀起全民创业热潮。抓好登记注册、税费减免、资金扶持、创业培训等政策落地，最大限度降低创业门槛；重点鼓励大中专毕业生、待就业人员等群体初始创业，推动企业家、个体户等群体二次创业，支持有成果、有技术、有创意等群体创新创业；面向全社会抓好典型讲好创业故事，支持群团组织及各类社会组织发挥引导创业作用，营造形成创业改变人生、创业实现价值、创业成就梦想的文化氛围。力促自主创业2万人以上，新增城镇就业4万人。兜住底线，提高社会保障水平。推动“五险”扩面新增1.8万人，提高企业退休人员和城乡居民基本养老金水平，推进四县医保市级统筹，新农合人均筹资标准提高到470元，实施城乡居民大病保险，推动省内异地就医即时结算；适时提高城乡低保标准，健全完善临时救助制度；完善农村“空巢”老人日间照料功能设施，推进城市社区老年人日间照料中心全覆盖；改造惠民苑等5个老居住区，继续加强保障房建设和农村泥草房改造。统筹城乡，促进公共服务均衡发展。教育重点优化资源配置，实施第二期学前教育三年行动计划，全市普惠性幼儿园比例达到80%，进一步解决好入托贵和入托难问题；扩大义务教育远程覆盖，加强乡村和城市薄弱学校网络终端建设，充分发挥400个优质资源共享教室作用；采取增加岗位津贴、优先评定职称等措施，推动师范院校毕业生到乡村学校任教；完善省级示范高中招生配额政策，改革中考和初中学业水平考试制度；实行职业教育贯通培养，拓展校企合作，力争毕业生一次就业率95%以上。医疗着力完善服务体系，推进四县医疗联合体建设，实现技术支援、分级诊疗、双向转诊；深化基层医疗机构一体化管理，人均基本公共卫生服务经费提高到40元，继续为乡镇卫生院配备医学本专科毕业生，充实乡镇医护力量；建好全科医生临床培养基地，扩大全科医生签约服务，推动“健康大庆行动”扎实开展。文化坚持提升服务品质与丰富基层生活并举，弘扬大庆精神铁人精神，唱响大庆幸福谣，凝聚价值共识，涵养文明风尚；鼓励话剧、舞蹈、工业版画等艺术创作，办好第二届“聆听经典”交响音乐季，打造特色文化精品；推动各类文体场馆向群众开放，新建中心村文体广场70个，办好“大庆阅读季”、“社区音乐会”、“广场文化”等群众性文化活动。强化举措，维护社会平安和谐。畅通群众诉求表达渠道，用好“大庆论坛”、“微大庆”等新媒体平台，让群众建议受到重视、得到回应；深入推进依法治访，坚持事要解决、属事属地相结合的原则，重点解决农民工工资拖欠、房地产开发、征地拆迁、房屋质量、农村资源发包、物业管理等信访问题，依托市群众工作中心建好省、市、县区三级远程视频接访系统，为多方联动解决信访问题创造条件；突出抓好危险化学品、公共聚集场所、道路交通、建筑施工和食品药品等重点领域安全监管，推进占压油气管线等专项治理，确保安全生产形势总体平稳；深入开展“打四黑除四害”专项行动，提高突发事件应急处置能力，强化“四位一体”防范机制，维护社会和谐安定。

　　（五）提升城乡功能品质。强化综合管理。着力加强规划管理，严格落实规划监管责任，建立全程规划监管机制和规划巡查制度，严禁随意变更规划，依法依规建设城市；着力加强质量管理，健全工程质量监管长效机制，严格项目立项和工程建设法定程序，严格落实项目法人责任制，严格规范整顿建筑、建材和监理市场，确保工程质量安全；着力加强环境管理，突出抓好城乡结合部、背街小巷、露天集市和乡容村貌等细部治理，加大市场化建设运营和管理城市力度，提升城市智能化管理水平；城区植树200万株，农村造林绿化8.5万亩，抓好秸秆综合利用，开展大气污染专项整治，确保空气质量保持较高水平。完善城乡基础设施。推动机场增开国际国内航线航点、哈齐城际客专如期开通，支持建设500千伏哈尔滨至大庆市区南部输变电工程，建好东城水厂二期、供热供水管网改造等项目；改造提升通村屯道路，扩大村屯巷道铺装，绿化亮化200个村屯，新建改造农村安全饮水工程340处，完善农村垃圾治理设施，村屯收集、乡镇转运、县集中处理模式覆盖率达到60%，有条件的村屯力争实现“五有”，建设美丽乡村。增强兴业创业承载功能。提升产业发展平台，高新区、经开区、肇州农业科技园区及省级园区在“七通一平”基础上完善公共技术、检验检测、金融服务、信息咨询、物流配送等功能，建设联想移动互联中心等大数据平台，增强产业综合承载能力；搭建创新创业平台，完善经开区中科创业园、市科技成果转化中心、东北石油大学科技园等载体，建设绿地大学科技园孵化器、恒基光伏科研孵化基地、联想青年创富中心等项目，在企业培育、融资担保、品牌打造等方面搭建创新创业生态圈，努力使大庆成为投资兴业、全民创业的热土。

　　（六）深化改革扩大开放。全面深化改革。继续抓好行政审批制度改革，完善事权下放后的监管服务机制，推行责任清单和负面清单，参照省政府行政审批模式优化流程设计，搭建网络审批平台；建立重点信访问题市、县区及相关企业会商解决机制，完善重点领域矛盾纠纷防范化解机制，积极探索依法治访和依法解决问题的有效办法；推进公共资源配置市场化改革，探索ppp模式、资产证券化和融资租赁等途径，引导社会资本参与城乡基础设施项目建设；抓好市属国资国企改革，重点对产业相近、行业相关的企业资产优化重组，推动由“管资产”向“管资本”转变，建立现代企业制度；深化社区改革，参照市区社区管理模式规范四县社区的工作机制、工作程序和工作标准，提升县域社区规范化建设水平；完善社会治理体系，创新居民自治实现形式，发展基层新型社会组织，支持重点行业协会建设，增强全社会自我管理、自我调节和自我发展能力。同时，继续深化文化体制和县级公立医院等项改革。深度扩大对外开放。围绕25条重点产业链加大招商引资力度，引进超亿元产业项目60个以上；保持俄油进口恒量，扩大石化、机电、食品等地方产品出口规模；用好用活产品制造、货物通关、交易活动可以三地分离政策，支持具备条件的地方企业与俄罗斯企业合作进行资源开发，谋划争取俄油俄气延伸加工，积极参加省里举办的第六届中俄文化大集和第二届中俄博览会，力争在农业种植、出口产品加工、旅游等领域合作实现更大突破；加强与国内外友好城市交往，拓展人文、科技、经贸等方面交流合作。

三、加强政府自身建设

　　新常态下推动大庆可持续发展，必须按照市委“讲政治、顾大局、在状态、守纪律”的要求，全面加强政府自身建设。

　　（一）严守政治纪律和政治规矩。全市各级政府工作人员要忠诚于党和人民，旗帜鲜明，立场坚定，认真贯彻落实中央和省委省政府的决策部署，坚持市委统一领导，严格遵循市委确定的六项工作原则，树立科学的政绩观；严格执行中央八项规定，认真落实市委市政府各项工作制度和规则；主动接受人大法律监督和工作监督、政协民主监督，认真办理人大代表建议和政协委员提案，广泛听取民主党派意见建议，支持工会、共青团、妇联等群团组织开展工作。

　　（二）深入推进依法行政。围绕建设法治政府，去行政化思维强法治化思维、去权力意识强规则意识，努力做到职权法授、程序法定、行为法限、责任法究；健全依法决策机制，认真执行“公众参与、专家论证、风险评估、合法性审查、集体讨论决定”的重大行政决策法定程序，完善政府法律顾问制度，发挥好市专家委等智库作用；严格规范行政执法行为，建立健全行政裁量权基准制度，重点规范征地补偿、征收迁建等行为，加大食品药品、安全生产、道路运输等关系群众切身利益的重点领域执法力度；坚持以公开为常态、不公开为例外原则，全面深化政务公开，落实政府新闻发言人制度，发挥政务公开网等平台作用，及时发布政务信息，促进政务运行阳光透明。

　　（三）整体优化发展环境。围绕更好地为油田服务、为招商引资服务、为本土企业和小微企业服务、为全民创业服务，保持现场办公、市级大会办和局内小会办常态化，推行行政服务指南“一本通”，发挥企业投诉中心功能，创造良好的政务环境；加强诚信政府、诚信组织、诚信企业建设，严厉打击影响市场经济秩序的不法行为，创造良好的法治环境；尊重企业市场主体地位，主动提供个性化服务，把产业发展政策落实到企业、把政策法规宣传到企业，营造全社会重商、亲商、护商氛围，创造良好的市场环境。

　　（四）转变作风狠抓落实。始终坚持“负责任地干事、干负责任的事”工作理念，深入基层与群众面对面交流，既听群众讲又讲给群众听，把百姓需求什么与政府该做什么能做什么结合起来，帮助群众排忧解难；突出干字当头，坚决克服“少干没啥事、多干怕出事”，等一等“不想干”、畏首畏尾“不敢干”等消极心态，用心想事、主动干事、努力成事，尤其是抓项目要研究问题、明晰路径，扶企业要因企施策、具体管用，对上争取要吃透政策、积极主动，做到扑下身子抓落实、困难面前敢担当；创新思维理念，不用老眼光看待新矛盾，不用老招法推动新实践，不用老标准对接新要求，力求在视野站位、方式方法、能力素质等方面适应新形势、实现新作为。

　　（五）切实加强廉政建设。强化制度刚性约束，严禁在资金分配使用、国有资产监管、政府投资和采购、资源配置、公共工程建设中暗箱操作，严禁政府公务人员插手工程招投标等经济活动，严禁在为企业和项目服务过程中吃拿卡要，对滥用权力、设租寻租、不作为乱作为等行为严肃问责；严格执行党风廉政建设责任制，落实“一岗双责”，把廉政建设与经济发展和业务工作同部署、同检查、同落实，做到两手抓、两促进；大力倡导勤俭务实，进一步节约政府开支，降低行政成本，坚决反对奢侈浪费。

　　各位代表，大庆面临的挑战前所未有，转型发展停顿不得、耽误不起。我们要在市委坚强领导下，团结依靠全市人民，弘扬大庆精神铁人精神，始终坚守大庆人的责任使命，始终保持大庆人的风范士气，始终鼓足大庆人的创业劲头，真抓实干，开拓进取，为推动大庆可持续发展而努力奋斗！
